# Structural analysis and epitope prediction of HCV E1 protein isolated in Pakistan: an in-silico approach

**DOI:** 10.1186/1743-422X-10-113

**Published:** 2013-04-10

**Authors:** Sobia Idrees, Usman A Ashfaq

**Affiliations:** 1 Department of Bioinformatics and Biotechnology, Government College University (GCU), Faisalabad, Pakistan

**Keywords:** Hepatitis C Virus, T cell epitope, B cell epitope, 3D structure prediction, Vaccine

## Abstract

**Background:**

HCV infection is a major health problem causing acute and chronic hepatitis. HCV E1 protein is a transmembrane protein that is involved in viral attachment and therefore, can serve as an important target for vaccine development. Consequently, this study was designed to analyze the HCV E1 protein sequence isolated in Pakistan to find potential conserved epitopes/antigenic determinants.

**Results:**

HCV E1 protein isolated in Pakistan was analyzed using various bio-informatics and immuno-informatics tools including sequence and structure tools. A total of four antigenic B cell epitopes, 5 MHC class I binding peptides and 5 MHC class II binding peptides were predicted. Best designed epitopes were subjected to conservation analyses with other countries.

**Conclusion:**

The study was conducted to predict antigenic determinants/epitopes of HCV E1 protein of genotype 3a along with the 3D protein modeling. The study revealed potential B-cell and T-cell epitopes that can raise the desired immune response against HCV E1 protein isolated in Pakistan. Conservation analysis can be helpful in developing effective vaccines against HCV and thus limiting threats of HCV infection in Pakistan.

## Introduction

Hepatitis C Virus infection is a global health problem affecting 270 million people worldwide [1]. According to the World Health Organization, liver cancer by HCV caused approximately 308,000 annual deaths in 2004 [2]. The number of HCV infected indviduals is increasing day by day, and there is variability in the prevalence reports of HCV in Pakistan but according to majority of studies, HCV is prevalent among 2.4-6.5% adults and among 0.44-1.6% of children [3]. From the prevalence analysis, clearly HCV genotype 3a is most common in Pakistan [4].

HCV is an RNA virus like dengue virus, West Nile virus and yellow fever virus belonging to the Flaviviridae family [5] and has a 9.5 kb genome with a positive-single stranded RNA that encodes a large polyprotein which is cleaved to produce four structural (Core, E1, E2 and P7) and six non-structural proteins (NS2, NS3, NS4A, NS4B, NS5A, NS5B). These viral proteins are liable for viral replication and various cellular functions [5-8]. Among HCV structural proteins, envelope proteins play the primary role in viral entry. HCV envelope protein 1 (E1) is a transmembrane glycoprotein having a C-terminal domain responsible for membrane association and membrane permeability changes [9]. E1 acts as a fusigenic subunit of the HCV envelope and contains 4–5 N-linked glycans. As it is known that the interaction of the virion with various cell receptors results in HCV infection [10,11]. Therefore, it is important to target virus envelope proteins to stop viral entry. Although there is not much knowledge available about E1, but it is thought to be involved in intra-cytoplasmic virus-membrane fusion. Currently, the standard of care is pegylated interferon (PEG-INF) with ribavirin; this therapy gives 50% sustained virological response in genotype 1 and 80% for genotype 2 and 3 [12,13]. One of the top priorities in HCV infection should be the development of more effective therapies by developing antiviral compounds for infected patients.

For designing effective inhibitors against envelope proteins, it is important to have knowledge of the epitopic regions/antigenic determinants of these glycoproteins. Bioinformatics analysis has opened new vistas to provide more insights into protein sequence and structural features. Both B-cell and T-cell epitopes/antigenic determinants are important in raising desired immune responses and the number of epitopes and modulation of immune recognition of antigens can be influenced by deglycosylation of viral glycoproteins [14]. This study was designed to perform immunoinformatic analysis on the HCV E1 glycoprotein isolated in Pakistan and to analyze antigenicity, hydrophobicity, surface accessibility and epitopic location of epitopes in HCV glycoprotein structure.

## Methods

### Protein retrieval and comparative modeling

The HCV E1 protein sequence was retrieved from NCBI protein database using the ID: ACN92051. It was ascertained that the three-dimensional structure of the protein was not available in Protein Data Bank (PDB). Therefore, the present study was designed to predict the 3D model and to predict epitopes of HCV E1 proteins isolated in Pakistan. Primary structure analysis was performed using the Protparam online tool. The parameters computed by ProtParam [15] included the molecular weight, theoretical pI, amino acid composition, atomic composition, extinction coefficient, estimated half-life, instability index, aliphatic index, and grand average of hydropathicity (GRAVY) and secondary structure analysis was done using various online servers. Structure template with PDB ID 2VOV_A having 43% identity was selected for the E1 protein. This template was used as a reference to determine the 3D structures of E1. Protein Structure Prediction Server (PS)^2^[16] predicted the homology model based on a package MODELLER. Moreover, Glycosylation sites of HCV E1 of Pakistani origin were found and their conservation with other regions of the world was also checked through Multiple Sequence Alignment. For this purpose, HCV E1 protein sequences isolated in different countries were retrieved from the NCBI protein database.

### Stereochemical analysis and model evaluation

Once the 3D model was generated, the Swiss-PdbViewer energy minimization test was applied to check for energy criteria in comparison with the potential of mean force derived from a large set of known protein structures. Structural evaluation and stereochemical analyses were performed using different evaluation and validation tools. Backbone conformation was evaluated by analyzing the Psi/Phi Ramachandran plot obtained from PROCHECK analysis. The Ramachandran plot of the phi/psi distribution in the model is developed using PROCHECK [17] for checking non-GLY residues at the disallowed regions. The Z-score is indicative of overall model quality and is used to check whether the input structure is within the range of scores typically found in native proteins of similar size. The Z-score was determined by PROSA web tool [18]. The model was further evaluated through ERRAT [19]. Furthermore, visualization of the generated model was performed using UCSF Chimera 1.5.3. The model generated for protein was successfully submitted to the Protein model database (PMDB) having PMID PM0078432.

### T-cell epitope and B-cell epitope prediction

A systemic strategy was adapted to design potential T-cell and B-cell epitopes of HCV envelope protein. VaxiJen v2. 0 online antigen prediction server was used for analyzing the antigenicity of the E1 protein [20]. Transmembrane topology of protein was checked using TMHMM [21]. B-cell epitopes were predicted using the BCPREDS online server using 75% of specific criteria for epitope prediction. All the predicted B-cell epitopes were checked from whether they were present in transmembrane regions or not using TMHMM results, and epitopes exposed on the surface of the membrane were selected and were subjected to further analysis. Antigenecity of selected epitopes were again checked using the Vexijen online server. DiscoTope server predicts discontinuous B-cell epitopes from protein three-dimensional structures. Disco Top 2.0 Server [22] was employed for discontinuous B-cell prediction using 3D structure of the HCV E1 protein of Pakistan. Furthermore, T-cell epitopes were screened. For this, Propred-1 which predicts epitopes for 47 MHC Class-I alleles and Propred, which predicts epitopes for 51 MHC Class-II alleles were utilized. Both servers cover a maximum number of HLA (Human Leukocyte antigens), therefore, are considered acceptable for predicting epitopes. Proteasome and immunoproteasome filters were set to a 5% threshold for MHC class I alleles. MHC binders that have proteosomal cleavage site at the C - terminal have greater chances to be T-cell epitopes [23].

### Epitope conservation analysis

Sequences of HCV E1 protein belonging to different regions of the world were retrieved from the NCBI database. A consensus sequence was drawn for each country, and all the consensus sequences were subjected to multiple sequence alignment using CLC workbench (data not shown). All the selected epitopes were checked for their conservation and variability by analyzing the multiple sequence alignment results and with the IEDB conservation analysis tool.

## Results

### Structural description of the model

**Figure 1:**
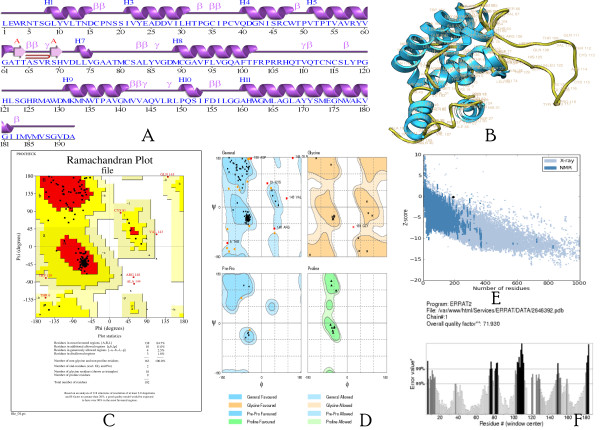
**A. Secondary structure of the HCV E1 protein of Pakistani origin, Helices are labeled as H1, H2; Beta turn as β; Gamma turn as χ and Beta hairpin as ⥰. B**. Predicted 3 Dimensional structure of the HCV Envelope protein 1 using Homology Modelling. **C**. Ramachandran plot showing residues in the most favorable region and disallowed regions. **D**. Z-score showing the quality of the 3D structure.

The present study was initiated to perform structure based sequence analysis studies on the HCV E1 protein isolated in Pakistan. The protein sequence was retrieved using accession #: ACN92051 from the NCBI protein database. Primary structure analysis showed that the E1 protein had a molecular weight of 20830.1 Daltons and theoretical isoelectric point (PI) of 6.62. An isoelectric point below 7 indicates a negatively charged protein. The instability index (II) is computed to be 21.17. This classifies the protein as stable. The N-terminus of the sequence is considered to be L (Leu). The negative Grand average of hydropathicity (GRAVY) of 0.316 indicated that the protein was hydrophobic. Valine (V), Glycine (G), Alanine (A) and Leucine (L) were found in rich amounts in the protein. Secondary structure revealed that it had 34.9% alpha helices, 8.8%, beta turns, 23.96% extended strand and 32.81% coils (Figure 1A).

Protein 3D structure is very important in understanding the protein interactions, functions and their localization [24]. Homology modeling is the most common structure prediction method. To perform the homology modeling, the first and basic step is to find a best matching template using similarity searching programs like PSI BLAST against a PDB database. Templates are selected based on their sequence similarity with query sequence. PDB ID 2VOV_A was selected for homology modeling, which is an X-ray diffraction structure of the Rev-erb Beta with resolution of 1.35 Å. Both template and target protein sequences were used to predict the 3D structure of the target protein using Protein Structure Prediction Server (PS) ^2^ (Figure 1B).

The 3D structure of the protein showed that it had 49 hydrogen bonds. Quality and reliability of structure were checked by several structure assessment methods, including Z-score, ERRAT and Ramachandram plots. Procheck checks the stereochemical quality of a protein structure by analyzing residue-by-residue geometry and overall structure geometry. This tool was used to determine the Ramachandran plot to assure the quality of the model. The result of the Ramachandran plot showed 84.5% of residues in the favorable region (Figure 1C, 1D). The Z-score is indicative of overall model quality and is used to check whether the input structure is within the range of scores typically found in native proteins of similar size. PROSA web was used to find the Z-score of the predicted structure. The Z - score of the protein was -0.11 (Figure 1E). Reliability of the model was further checked by ERRAT, which analyzes the statistics of non-bonded interactions between different atom types and plots the value of the error function versus position of a 9-residue sliding window, calculated by a comparison with statistics from highly refined structures. Results from ERRAT showed 71.930 overall model quality (Figure 1F). The Z-scores, Ramachandran plot and ERRAT results confirmed the quality of the homology model of the HCV E1 protein.

### Glycosylation site analysis

**Figure 2:**
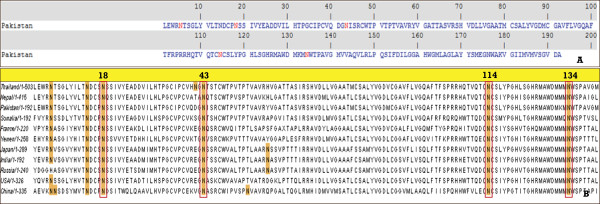
HCV EI protein glycosylation sites. **A**. The HCV E1 protein of Pakistani origin showing 5 glycosylation sites highlighted in red at positions 5, 18, 43, 114 and 134. **B**. Multiple sequence alignment showing conserved glycosylation sites at positions 5, 18, 43, 114 and 134 in the HCV E1 proteins isolated from the different region of the world.

N-glycosylation sites were searched in the HCV E1 protein sequence using criteria as Asn-X-Ser or Asn-X-three sequences, where X is any amino acid residue. Four glycosylation sites were found at position 5, 18, 43, 114 and 134 (Figure 2A). To find a conserved glycosylation site in an HCV E1 protein of other countries, a multiple alignment using CLUSTALW was performed, and it was found that all glycosylation sites at position 5, 18, 43, 114 and 134 were conserved in E1 proteins of different countries as well as with Pakistan (Figure 2B).

### Epitope prediction

Overall antigenicity of E1 protein was predicted to be 0.5362 indicating it as a probable antigen. Transmembrane protein topology was checked using the TMHMM online tool, and was found that residues 1–73 presented outside while residues 74–96 were within the transmembrane region, and residues 97–192 were inside the core region of the protein.

### B-cell epitope prediction

**Figure 3:**
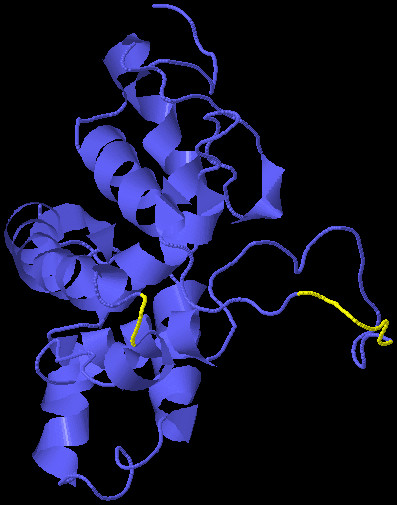
**Predicted B-cell epitopic regions of the E1 protein 3D structure. **B-cell epitopic regions are shown in yellow color.

B-cell epitopes are important for protection against virus infection. B-cell epitope prediction was performed using BCPRED server where criteria were set to have 75% specificity and 12 aa epitope length [25]. A total of six B-cell epitopes were predicted using a BCPRED server (Table 1). After checking the TMHMM results, it was found that epitope VGQAFTFRPRRH, with 0.538 BCPred score was in the transmembrane region while epitopes TPVTPTVAVRYV, TPGCIPCVQDGN, TNDCPNSSIVYE with 0.994, 0.965 and 0.87 scores, respectively, were exposed outside. Antigenecity of VGQAFTFRPRRH epitope was found to be 0.8539 and antigenicity of exo-membrane epitopes were 1.1421 for TPVTPTVAVRYV, 0.9738 for TPGCIPCVQDGN indicating these epitopes as probable antigens while the antigenic score of TNDCPNSSIVYE was 0.2295 indicating it as a non-antigen, thereby, resulting in its exclusion. From the results, it can be inferred that these epitopes/antigenic determinants are important in raising the desired immune response. Moreover, the 3D structure of E1 was used to predict conformational discontinuous B-cells epitopes using the Disco Top 2.0 online server. A total of 8 B-cell epitopic locations were found from the 3D structure of the protein (Table 2). B-cells epitopes are shown in yellow color in the 3D structure of the E1 protein Figure 3.

**Table 1:** Predicted B-cell epitopes

**Position**	**Epitope**	**Score**
49	TPVTPTVAVRYV	0.994
32	TPGCIPCVQDGN	0.965
13	TNDCPNSSIVYE	0.87
153	IFDILGGAHWGM	0.702
96	VGQAFTFRPRRH	0.538
123	SGHRMAWDMKMN	0.292

**Table 2:** Discontinuous epitopes predicted from the 3D structure of the E1 protein using DiscoTop online server

**Position**	**Residue**	**Contact number**	**Propensity score**	**DiscoTope score**
109	THR	3	-2.545	-2.598
110	VAL	7	-2.183	-2.737
111	GLN	3	-1.235	-1.438
112	THR	1	-1.964	-1.853
113	CYS	1	-3.211	-2.957
114	ASN	9	-2.677	-3.404
155	ASP	1	-2.341	-2.187
156	ILE	1	-1.626	-1.554

### T-cell epitope prediction

**Figure 4:**
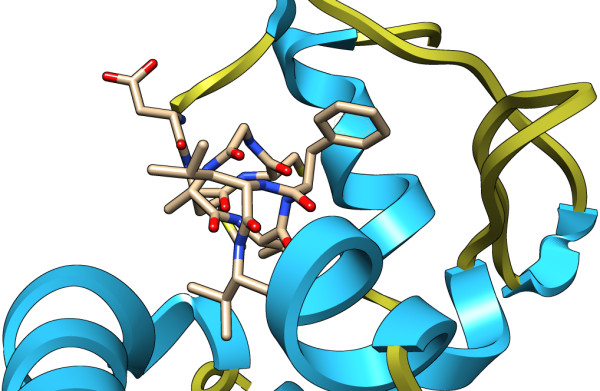
The HCV E1 protein model showing an epitopic location in the structure.

Propred-I (47 MHC Class-I alleles) and Propred (51 MHC Class-II alleles) were used to predict T-cell epitopes for the HCV E1 protein. ProPred1 is an online web tool for the prediction of peptides binding to MHC class-I alleles. The HCV E1 sequence was uploaded to the Propred server while selecting all the alleles, with a high scoring peptide threshold of 4%, and showing the top four epitopes in the tabular form along with proteasome and immunoproteasome filters. All the predicted epitopes were checked for their antigenicity and epitopes that were found to be antigenic in nature were used for further analysis (Table 3). Epitope MNWTPAVGM at position 154 was found to have the highest antigenicity among all epitopes assuring maximum binding affinity. The HCV E1 sequence was also used to predict MHC class II binding regions using the Propred online server (Table 4). Epitope YVGATTASV at position 30 was found to have the highest antigenicity ensuring maximum binding affinity. The HCV E1 protein structure with an epitope selected is shown in (Figure 4).

**Table 3:** MHC class I binding peptides on the basis of antigenicity

**Starting position**	**Peptide**	**Allele**	**Antigenic score**
83	ATTASVRSH	HLA-A1/HLA-A*1101/HLA-A3/HLA-A*3101/HLA-A*3302/HLA-B*5801	0.9061
154	MNWTPAVGM	HLA-A2/HLA-A*0201/HLA-A*3101/HLA-A20/HLA-B*2705/HLA-B*3501/HLA-B*5201/HLA-B*5301/HLA-B*5401/HLA-B*51/HLA-B7/HLA-B*0702/HLA-B8/HLA-Cw*0401/MHC-Ld	1.1593
160	VGMVVAQVL	HLA-A*0205/HLA-A24/HLA-A20/HLA-A2.1/HLA-B14/HLA-B*2702/HLA-B*2705/HLA-B*3701/HLA-B*3801/HLA-B*3901/HLA-B*3902/HLA-B*4403/HLA-B*5101/HLA-B*5102/HLA-B*5103/HLA-B*5201/HLA-B*5301/HLA-B*51/HLA-B60**/**HLA-B62/HLA-B7/HLA-B8/HLA-Cw*0301/HLA-Cw*0401/HLA-Cw*0602/HLA-Cw*0702/MHC-Db/MHC-Dd/MHC-Kb**/**MHC-Kd**/**MHC-Kk/MHC-Ld	0.5535
178	LGGAHWGML	HLA-A*0205/HLA-A24/HLA-A2.1/HLA-B14/HLA-B*2705/HLA-B*3701/HLA-B*3901/HLA-B*3902/HLA-B*5101/HLA-B*5102/HLA-B*5103/HLA-B*5201/HLA-B*51/HLA-B60/HLA-B62/HLA-B7/HLA-B*0702/HLA-Cw*0301/HLA-Cw*0401/HLA-Cw*0602/HLA-Cw*0702/MHC-Db/MHC-Dd/MHC-Kb/MHC-Kd	0.6771
90	SHVDLLVGA	HLA-A*3302/HLA-B*3801/HLA-B*3901/HLA-B*5401/HLA-Cw*0702	0.9645

**Table 4:** MHC class II binding peptides on the basis of antigenicity

**Starting position**	**Peptide**	**Allele**	**Antigenic score**
12	LTNDCPNSS	DRB1_0305-309, DRB1_0311, DRB1_0401, DRB1_0421, DRB1_0426, DRB1_1107	0.4554
19	WTPVTPTVA	DRB1_0101, DRB1_408	0.7528
28	VRYVGATTASV	DRB1_0101, DRB1_0305, DRB1_0309, DRB1_0402, DRB1_0404, DRB1_0405, DRB1_0408, DRB1_0410, DRB1_0423, DRB1_0813, DRB1_1107	0.5239
30	YVGATTASV	DRB1_0101, DRB1_0305, DRB1_0309, DRB1_0401, DRB1_0402, DRB1_0404, DRB1_0405, DRB1_0408, DRB1_0410, DRB1_0421, DRB1_0423, DRB1_0426, DRB1_0701, DRB1_0703, DRB1_0801, DRB1_0802, DRB1_0813, DRB1_1101, DRB1_1114, DRB1_1120, DRB1_1128, DRB1_1302, DRB1_1305, DRB1_1307, DRB1_1321, DRB1_1323	1.0175
28	VRYVGATTA	DRB1-0102, DRB1-0306-0308, DRB1_0311, DRB1_1104, DRB1_1106, DRB1_1107, DRB1_1311, DRB1_1501, DRB1_1506, DRB5_0101, DRB5_0105	0.4463

### Epitope conservation and variability analysis

Moreover, the conservation of all predicted epitopes was checked by analyzing and comparing all the epitope sequences of the HCV E1 protein with E1 of other regions of the world. E1 sequences used in this study were from Somalia (AAF44733.1), Nepal (BAA04038.1), Canada (ABI23143.1), China (AAK95634.1), Japan (BAD06555.1), France (CAJ45644.1), India (AAG09116.1), Russia (CAD44972.1), USA (AAD21251.1) and Yemen (BAA07778.1) and were used for comparative studies through multiple alignment using ClustalW followed by verification with IEDB epitope conservation analysis resource [26]. Conservation analysis of epitopes showed conserved and variable residues of epitopes in the E1 sequences of other countries, and it was found that most of the predicted epitopes were conserved with the E1 sequence of Canada while having some conservation with other countries as well (Table 5).

**Table 5:** Conservation and variability analysis of B-cell and T-cell epitopes in comparison with HCV E1 proteins of other regions

**Peptide**	**India**	**Russia**	**Japan**	**USA**	**China**	**Nepal**	**Yemen**	**France**	**Canada**	**Somalia**
VGQAFTFRPRRH	V**S**Q**L**FTF**S**PRRH	V**S**Q**L**FTF**S**PRRH	**IS**Q**L**FTF**S**PRRH	VGQ**L**FTF**S**PR**H**H	**AA**Q**L**F**IIS**PXH**H**	VGQAFTF**S**PRRH	VGQ**VI**TF**K**PRRH	VGQ**M**FT**Y**RPR**Q**H	VGQAFTFRPRRH	VGQAF**R**FR**Q**R**Q**H
TPVTPTVAVRYV	**VAL**TPT**L**A**A**R**NA**	**VAL**TPT**L**A**A**R**NA**	**VAL**TPT**L**A**A**R**NS**	**VA**V**A**PTVA**T**R**DG**	**I**PV**S**P**NI**AV**QQP**	TPV**S**PTVAV**KHL**	**K**PVTPTVAV**A**Y**G**	**VQI**TPT**LSAPSF**	TPVTPTVAVRYV	TPVTPTVAVR**AP**
TPGCIPCVQDGN	TPGC**V**PCV**RE**GN	TPGC**V**PCVQ**ED**N	TPGC**V**PCV**RE**GN	**S**PGC**V**PCV**RE**GN	**V**PGC**V**PC**EKV**GN	**L**PGC**V**PCV**ATA**N	**L**PGC**V**PCV**KT**GN	TPGC**V**PCV**KE**GN	TPGCIPCVQDGN	**S**PGC**V**PCV**KS**GN
TNDCPNSSIVYE	TNDC**S**NSSIVYE	TNDC**S**NSSIVYE	TNDC**S**NSSIVYE	TNDCPNSSIVYE	TNDC**S**N**D**S**ITWQ**	TNDC**S**N**Q**SIVYE	TNDCPNSS**V**VYE	TNDCPNSSIVYE	TNDCPNSSIVYE	TNDCPNSSIVYE
ATTASVRSH	**VP**T**TTIRR**H	**VP**T**TAI**R**R**H	**VP**T**TTI**R**R**H	**LP**T**TQL**R**R**H	A**L**T**RGL**R**T**H	ATTAS**I**RSH	A**PLE**S**F**R**R**H	A**X**TA**PL**R**R**A	ATTASVRSH	**VI**TAS**I**RSH
MNWTPAVGM	MNW**S**P**TAAL**	MNW**S**P**TTAL**	MNW**S**P**TAAL**	MNW**S**P**TTAL**	MNW**S**P**TAT**M	MNW**S**PA**IGL**	MNW**S**P**TTTL**	MNW**S**P**TTAL**	**N/A**	**Q**NW**S**P**T**V**SL**
VGMVVAQVLRL	**AAL**VV**S**Q**L**LR**I**	**TAL**VV**S**Q**L**LR**I**	**AAL**V**AS**Q**LF**R**I**	**TAL**VVAQ**LL**R**V**	**AT**M**IL**A**YAM**R**I**	**I**G**LA**V**SHLM**RL	**TTLLL**AQ**IM**R**I**	**TALLM**AQ**L**LR**I**	**N/A**	V**SLI**VAQVLRL
LGGAHWGML	**VA**GAHWG**I**L	**VA**GAHWG**V**L	**VA**GAHWG**V**L	**IA**GAHWG**V**L	**IS**GAHWG**VM**	**IA**GAHWG**VM**	**VA**G**G**HWG**V**L	**VA**G**G**HWG**V**L	**N/A**	L**V**G**S**HWG**V**L
SHVDLLVGA	**R**HVDLLVGA	**R**HVDLLVGA	**R**HVDLLVGA	**R**H**I**DLLVG**S**	**T**H**I**D**MV**V**MS**	SHVD**M**LVGA	**R**HVDL**M**VGA	**RA**VD**Y**L**A**G**G**	SHVDLLVGA	SHVDL**M**VG**S**
LTNDCPNSS	**V**TNDC**S**NSS	**V**TNDC**S**NSS	**V**TNDC**S**NSS	**V**TNDCPNSS	**V**TNDC**S**N**D**S	LTNDC**S**N**Q**S	**I**TNDCPNSS	**V**TNDCPNSS	LTNDCPNSS	**V**TNDCPNSS
WTPVTPTVA	W**VAL**TPT**L**A	W**VAL**TPT**L**A	W**VAL**TPT**L**A	W**VA**V**A**PTVA	W**I**PV**S**P**NI**A	WTPV**S**PTVA	W**K**PVTPTVA	W**VQI**TPT**LS**	WTPVTPTVA	WTPVTPTVA
VRYVGATTASV	**A**R**NASVP**T**TTI**	**A**R**NASVP**T**TAI**	**A**R**NSNVP**T**TTI**	**T**R**DGKLP**T**TQL**	V**QQP**GA**L**T**RGL**	V**KHL**GATTAS**I**	V**A**YG**S**A**PLE**S**F**	**APSF**GA**X**TA**PL**	VRYVGATTASV	VR**AP**G**VI**TAS**I**
YVGATTASV	**NASVP**T**TTI**	**NASVP**T**TAI**	**NSNVP**T**TTI**	**DGKLP**T**TQL**	**QP**GA**L**T**RGL**	**HL**GATTAS**I**	Y**GS**A**PLE**S**F**	**SF**GA**X**TA**PL**	YVGATTASV	**AP**G**VI**TAS**I**
VRYVGATTA	**A**R**NASVP**T**T**	**A**R**NASVP**T**T**	**A**R**NSNVP**T**T**	**T**R**DGKLP**T**T**	V**QQP**GA**L**T**R**	V**KHL**GATTA	V**A**Y**GS**A**PLE**	**APSF**GA**X**TA	VRYVGATTA	VR**AP**G**VI**TA

Variable residues in epitopes are shown in bold.

## Discussion

In this study, sequence and structure analysis, homology modeling and epitope analysis was performed on the HCV E1 protein isolated in Pakistan. We have used various sequence and structure analysis tools that helped in understanding of the sequence and its structure. Through primary structure analysis, amino acid composition of the HCV E1 glycoprotein was checked, and it showed that it has maximum Valine (V) residues and its N-terminus is a Leucine (L).

We used a homology modeling approach to predict the 3D structure of the HCV E1 protein of Pakistan. The predicted 3D structure will provide more insight into understanding the structure and function of this protein. Moreover, this structure can be used for drug designing or understanding the interactions between proteins. The HCV E1 protein was molecularly characterized using various online servers, and it was observed that it had five glycosylation sites, and all of them were conserved in HCV E1 protein sequences of other countries. Clustal W multiple sequence alignment was used to determine the conservation and variability of HCV E1 protein belonging to different regions of the world, and it was determined that there were frequent variations at position 6 (Threonine), 11 (Valine), 17 (Proline), 32 (Threonine), 36 (Isoleucine), 40 (Glutamine), 41 (Aspartic Acid), 44 (Isoleucine), 45 (Serine), 46 (Arginine), 50 (Proline), 58 (Arginine), 59 (Tyrosine), 62 (Alanine), 67 (Valine), 77 (Alanine), 89 (Metheonine), 96 (Valine), 103 (Arginine), 116 (Serine), 123 (Serine), 132 (Lysine), 136 (Threonine), 144 (Alanine), 145 (Glutamine), 152(Serine), 153 (Isoleucine), 157 (Leucine), 158 (Glutamine), 164 (Metheonine), 174 (Glutamic Acid), 181 (Glutamine), 182 (Isoleucine), 185 (Valine), 187 (Valine) in the HCV E1 protein sequences. All other residues of the HCV E1 protein were conserved in all sequences.

As a part of the present study, we predicted B-cell and T-cell epitopes of the HCV E1 protein using different online tools. Only four B-cell epitopes were found to be antigenically effective, and it can be inferred that these epitopes/antigenic determinants are important in raising the desired immune response. Using 3D structure of the E1 protein, eight B-cell epitopic locations were identified. All the predicted B-cell epitopes were checked for their localization in the protein structure, and it was found that the majority of predicted epitopes were in the outside region of the protein. T-cell epitopes were predicted using Propred I and Propred online servers and their antigenicity was found using the Vexijen online server. It was found that the MHC class I binding peptide MNWTPAVGM and the MHC class II binding peptide YVGATTASV had maximum antigenecity ensuring maximum binding affinity. Furthermore, all the selected epitopes were checked for their conservation with other countries of the world, and it was found that most of the epitopes were conserved among Pakistan and Canada, suggesting that these E1 epitopes of these two countries may be evolutionary related. Moreover, all the epitopes showed some conservation with all other countries but there were frequent variations at some points.

## Conclusion

To develop effective vaccines it is important to target multiple antigenic components of the virus, thus directing the immune system to protect the host from the virus. Therefore, this study was conducted to predict antigenic determinants/epitopes of the HCV genotype 3a E1 protein along with the 3D protein modeling. The study revealed potential B-cell and T-cell epitopes that can raise the desired immune response to the HCV E1 protein isolated in Pakistan. For diagnosing HCV genotype 3a, these epitopes are highly useful and can also help in developing successful vaccines against HCV 3a infection to save the Pakistani population from potential HCV threats.

## Competing interests

All authors have no institutional or financial competing interests.

## Authors’ contributions

UAA designed the study, and SI wrote the manuscript. UAA and SI performed all in-silico work, and UAA critically reviewed the manuscript. All the authors read and approved the final manuscript.

## Authors’ information

Sobia Idrees (MPhil student), Usman A Ashfaq (PhD molecular Biology and Group leader, Human Molecular Biology Group, Department of Bioinformatics and Biotechnology, GCU, Faisalabad.
